# Determinants of success and failure of antibody-based strategies against respiratory viruses: insights from RSV and SARS-CoV-2

**DOI:** 10.3389/fimmu.2026.1818721

**Published:** 2026-05-01

**Authors:** Hai Guo, Hongshu Wang, Shutong Wu, Haonan Lin, Guosong Wang, Qiang Pu

**Affiliations:** 1 Department of Thoracic Surgery, West China Hospital, Sichuan University, Chengdu, Sichuan, China; 2 Department of Thoracic Surgery, Chuxiong Prefecture People’s Hospital, Chuxiong, Yunnan, China; 3 Department of Experimental Research, Sichuan Clinical Research Center for Cancer, Sichuan Cancer Hospital & Institute, Sichuan Cancer Center, University of Electronic Science and Technology of China, Chengdu, Sichuan, China

**Keywords:** monoclonal antibodies, mucosal immunity, respiratory syncytial virus, SARS−CoV−2, viral evolution

## Abstract

Respiratory syncytial virus (RSV) and severe acute respiratory syndrome coronavirus 2 (SARS−CoV−2) represent two extremes in the outcome of antibody−based interventions. The long−acting monoclonal antibody nirsevimab has achieved durable, population−level protection against RSV in infants, reducing hospitalizations by 70–90% with no evidence of antigenic escape. In contrast, all neutralizing monoclonal antibodies against SARS−CoV−2 became obsolete within three years due to rapid viral evolution, particularly in the spike receptor−binding domain. This review dissects the mechanistic determinants underlying this divergence. We propose four key principles that govern antibody efficacy against respiratory viruses: (i) targeting a structurally conserved epitope with high fitness cost for escape; (ii) achieving sufficient antibody concentrations in the airway epithelial lining fluid; (iii) the vulnerability of single−epitope strategies against mutable viral targets; and (iv) the auxiliary but non−substitutable role of Fc effector functions. By comparing RSV and SARS−CoV−2, we illustrate how these principles align in successful interventions and fail in others. Finally, we discuss emerging strategies—particularly inhaled delivery and mRNA−encoded antibodies—that may overcome current limitations and enable durable protection against antigenically variable respiratory pathogens.

## 1 Introduction

Respiratory viral infections remain a leading cause of global morbidity and mortality, especially among infants, older adults, and immunocompromised populations (1–3). Respiratory syncytial virus (RSV) and severe acute respiratory syndrome coronavirus 2 (SARS−CoV−2) each cause tens of millions of infections and substantial mortality annually, particularly in infants, older adults, and high−risk groups (4, 5). RSV is responsible for 4.7-7.8% of respiratory infections in older and high−risk adults, with a fatality rate of 8-10% (6), in hospitalized older adults, it frequently leads to severe lower respiratory tract disease associated with a 5.6% in−hospital and 25.8% one−year mortality (7). SARS−CoV−2 has a case fatality rate exceeding that of influenza, and the B.1.1.7 variant increased mortality by 64% compared to prior variants (8, 9).

Despite decades of antiviral development, effective therapeutics are limited, and vaccines often show waning immunity and incomplete mucosal protection (10–14). Monoclonal antibodies (mAbs) emerged as an ideal countermeasure: they provide immediate, highly specific protection by targeting conserved viral epitopes (15–19). However, real−world outcomes have diverged dramatically between viruses (20).

RSV has become the first respiratory pathogen for which antibody prophylaxis achieved a population-level impact (21–23). The long−acting mAb nirsevimab reduced RSV lower respiratory tract infections by 70–80% in trials, and real−world data from the 2024–2025 season confirm 80–90% reductions in infant hospitalizations, with no evidence of antigenic escape (21, 24–27). In contrast, SARS−CoV−2 became the first virus to eliminate an entire therapeutic class within a few years (28). During 2020–2021, neutralizing antibodies (e.g., REGEN−COV, sotrovimab, Evusheld) provided significant clinical benefits (29–32). but beginning with Omicron in late 2021 and accelerating through XBB and JN.1 sublineages, the virus accumulated spike RBD mutations that rendered every therapeutic antibody ineffective (33–39). An estimated multi−billion−dollar investment was lost within three years (40).

This striking divergence poses a fundamental question: Why do antibody−based interventions succeed for some respiratory viruses yet fail completely for others? Answering this requires integrating viral evolution, epitope structure, mucosal pharmacokinetics, and immune effector mechanisms. RSV and SARS−CoV−2 serve as natural experimental opposites that reveal the mechanistic circumstances under which antibodies can—and cannot—work. In this review, we dissect these determinants and propose four mechanistic principles that govern antibody efficacy.

## 2 Mechanistic determinants of antibody success or failure

For over a century, polyclonal and monoclonal antibodies have been employed in the treatment and prevention of infectious diseases. Neutralizing antibodies confer protection against respiratory viruses through two integrated layers of antiviral activity: direct Fab−mediated neutralization of virions and Fc−dependent effector functions that eliminate infected cells and augment viral clearance (41, 42).

The primary mechanism of antibody-mediated protection involves preventing viral entry into host cells. Antibodies achieve this by binding to viral surface proteins—envelope glycoproteins for enveloped viruses or capsid proteins for non-enveloped viruses—that govern two essential functions: receptor engagement and membrane fusion (or cytoplasmic penetration) (43). For SARS−CoV−2, entry is mediated by the spike (S) glycoprotein binding to angiotensin-converting enzyme 2 (ACE2) receptors expressed on respiratory, gastrointestinal, and endothelial cells (44, 45). Antibodies targeting the spike receptor-binding domain (RBD) sterically occlude this interaction, thereby blocking infection at the earliest stage (46, 47). For RSV, antibodies targeting the prefusion F protein can interfere with both receptor tethering (e.g., to IGF1R or nucleolin) and the conformational rearrangements required for membrane fusion (19, 48, 49).

**Figure 1:**
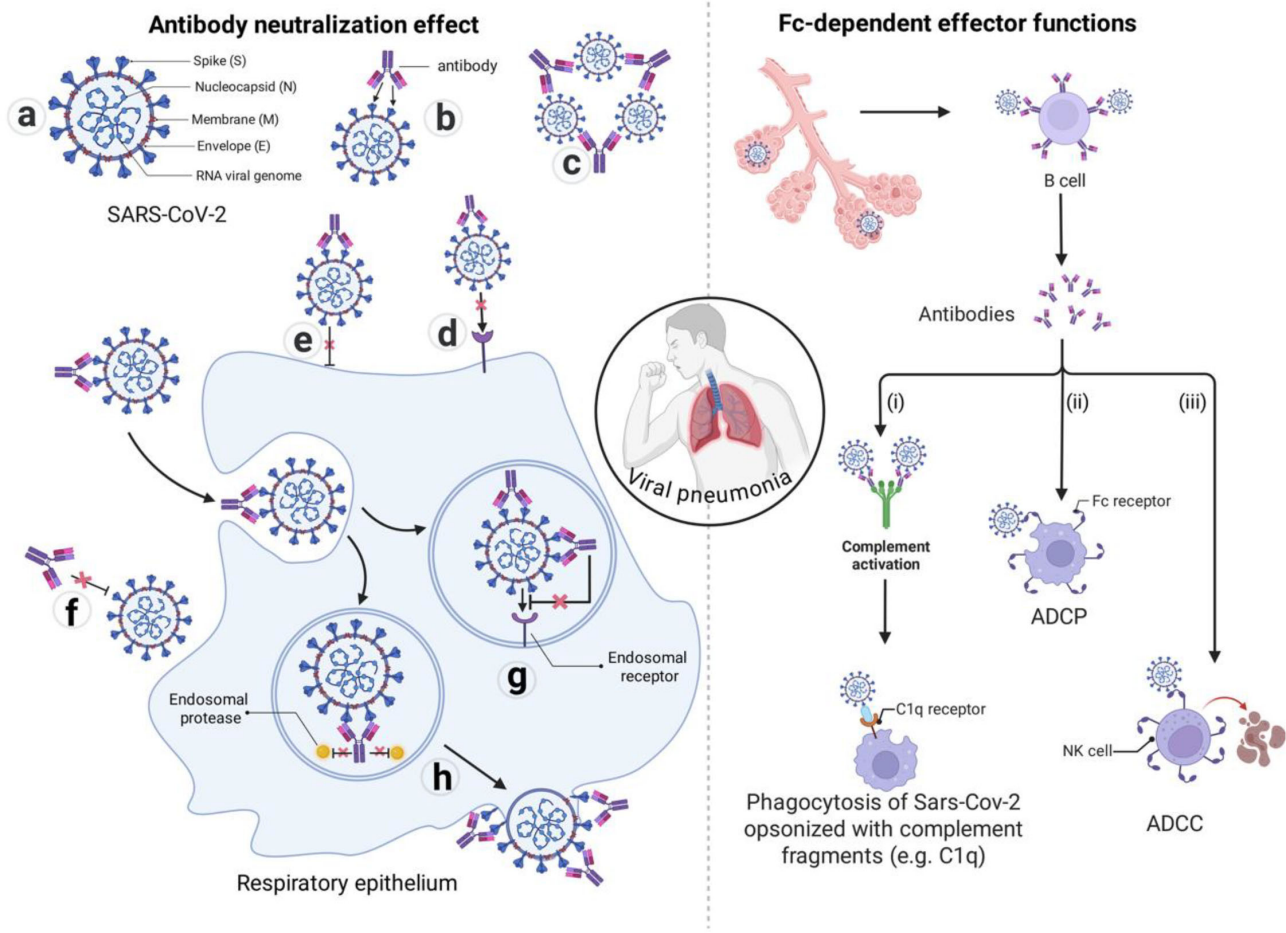
Mechanisms of antibody-mediated immunity against respiratory viruses (using SARS-CoV-2 as an example). The left side illustrates the mechanisms of direct virus neutralization, while the right side depicts Fc-dependent antiviral activities. (Left) Direct neutralization: **(a)** Schematic structure of the SARS-CoV-2 virus. **(b)** Antibody-mediated dissociation or conformational modification of the spike protein, preventing virion attachment to the host cell receptor. **(c)** Antibody-induced virion aggregation, which may contribute to neutralization by reducing the number of particles able to bind to target cells. **(d)** Steric hindrance by antibodies directly blocking the interaction between the spike protein and the host cell receptor. **(e)** Steric hindrance by antibodies blocking the fusion of the viral envelope with the host cell membrane. **(f)** Antibodies blocking the conformational changes in the spike protein required for viral entry. **(g)** For viruses entering via the endosomal pathway, antibodies can block entry into the cytoplasm by inhibiting endosomal cleavage and/or endosomal receptor binding. **(h)** Antibodies inhibiting the release of progeny virus from an infected cell.(Right) Fc-mediated mechanisms: In the presence of complement and/or effector cells: **(i)** Antibody-coated virions are subject to enhanced neutralization and complement-mediated lysis. **(ii)** In the presence of FcR, Fc receptor bearing effector cells, antibody-coated virions can be opsonized and prevented from binding to host cells, or they can be taken up by phagocytes such as macrophages via ADCP, antibody-dependent cellular phagocytosis. **(iii)** Virus-infected cells coated with antibodies can be recognized and killed by effector cells such as NK, natural killer cells via FcR-dependent, ADCC, antibody-dependent cellular cytotoxicity. Image created with Biorender.com, with permission.

Beyond direct neutralization, antibodies engage the immune system through their Fc domains. Binding of antibodies to viral antigens can recruit complement protein C1q, initiating the classical cascade that leads to direct lysis of virions or infected cells (complement-dependent cytotoxicity, CDC) (50, 51). Simultaneously, engagement of Fcγ receptors on leukocytes—including natural killer cells, macrophages, neutrophils, and monocytes—triggers antibody-dependent cellular cytotoxicity (ADCC), antibody-dependent cellular phagocytosis (ADCP), or release of cytotoxic mediators such as cytokines and reactive oxygen species (50, 52–54). These effector mechanisms enable elimination of infected cells before progeny virions are released and enhance clearance of opsonized viral particles (**Figure 1**).

Antibody performance in the respiratory tract is governed by predictable biological constraints. The contrasting outcomes of RSV and SARS−CoV−2 antibody interventions can be explained by four key determinants, summarized and discussed (**Table 1**).

**Table 1:** Key mechanistic determinants of antibody afficacy against respiratory viruses: comparison between RSV and SARS-CoV-2.

Determinant	RSV	SARS-CoV-2
Epitope conservation	Prefusion F site Ø is structurally constrained, highly conserved, and mutations impose high fitness cost.	RBD is highly mutable, under intense immune selection; extensive antigenic drift.
Mucosal antibody concentration	Low concentrations sufficient due to high mAb potency and slower viral replication.	Rapid, high-titer replication outpaces systemic IgG penetration.
Single-epitope vulnerability	Site Ø rigidity limits escape; no persistent resistance observed post-licensure.	Single RBD epitopes easily mutated; cocktails also overcome by concurrent substitutions.
Fc effector function contribution	Enhances protection but neutralization is primary; Fc alone insufficient.	Fc functions augment but cannot rescue lost neutralization; essential for vaccine-elicited protection against shifted variants.

### 2.1 Viral evolutionary rate and epitope conservation

Durable efficacy requires targeting an antigenically stable epitope. RSV’s prefusion F protein, especially the apex “site Ø” targeted by nirsevimab, is structurally constrained and highly conserved (55–57). Escape mutations at this site impose a high fitness cost; indeed, decades of RSV surveillance have observed essentially no persistent mutations in site Ø (58). By contrast, the SARS-CoV-2 spike receptor-binding domain (RBD) is extremely mutable and under intense immune selection (59, 60). Within two years, Omicron−lineage viruses accumulated over 30 amino acid substitutions in the RBD, enabling evasion of every known neutralizing mAb (61–63). Epistatic mutation networks allowed SARS-CoV-2 to rapidly adapt and escape even multi-antibody cocktails (64). For example, recurrent substitutions at Spike L452 and F490 undermined a public VH1–69 antibody response, demonstrating how single-site changes can drive population-level escape (65, 66). Viruses with antigenic evolution are unlikely to be durably controlled by a monoclonal antibody.

### 2.2 Mucosal concentration threshold in the epithelial lining fluid

Systemically delivered IgG achieves only a tiny fraction of its serum concentration in the airway mucosa, often insufficient to rapidly neutralize fast-replicating viruses (67, 68). Respiratory viruses replicate on airway epithelial surfaces, but intravenous antibodies distribute poorly to this compartment, and pharmacokinetic studies consistently demonstrate this compartmental mismatch (68–70). In mice, trastuzumab shows a bronchoalveolar lavage (BAL) exposure of approximately ~1/50 (≈2%) of plasma levels, with BAL concentrations 5–7 times lower than total lung tissue—highlighting that epithelial lining fluid (ELF) levels are substantially lower than tissue homogenate measurements suggest (70). In systemically dosed adults, the RSV−neutralizing mAb clesrovimab achieves a nasal ELF−to−serum ratio ranging from ~1:69 to 1:30 (≈1.4-3.3%) (71). Together, these data indicate that steady−state ELF concentrations of systemically administered mAbs typically reach only about 1–3% of concurrent plasma levels.

Such levels can protect against RSV, which replicates relatively slowly and can be neutralized at low concentrations. In contrast, SARS-CoV-2 replicates to high titers in the upper airway within days, reaching >10^7^ viral copies/mL before systemic IgG can accumulate in sufficient quantity (72–74). Effective antibody−mediated protection requires achieving local concentrations that exceed the neutralization threshold at the mucosal site (75). Inhaled delivery bypasses this limitation: in a hamster model, intratracheal delivery of a SARS−CoV−2 neutralizing mAb at a lung−deposited dose of only 0.03 mg/kg reduced pulmonary and upper respiratory viral loads to undetectable levels (76); in a Phase I trial, a single 90 mg nebulized dose of regdanvimab achieved median nasal fluid concentrations of ~740 µg/mL at 30 minutes, remaining at ~1.2 µg/mL after 22 hours-well above the *in vitro* neutralization IC_90_ (77). Thus, the ELF threshold rule: if standard dosing fails to sustain neutralizing antibody in the respiratory ELF at the onset of infection, reliable clinical protection is unlikely.

### 2.3 Single-epitope dependency is viable only for structurally rigid targets

Most therapeutic mAbs target a single dominant epitope. This approach can be effective when the epitope exhibits structural rigidity, limiting the virus’s tolerance to mutations without compromising fitness (56, 58). Nirsevimab binds to site Ø on the RSV prefusion F protein, a region highly conserved and functionally critical for membrane fusion; mutations here may impair fusogenicity, contributing to sustained susceptibility across strains (24, 78, 79). Recent surveillance data indicate that widespread immune−escape variants fully resistant to nirsevimab have not emerged, though rare substitutions conferring partial resistance have been observed in some RSV−B strains (80, 81). In contrast, neutralization epitopes on the SARS−CoV−2 RBD demonstrate greater structural plasticity, permitting mutations that preserve infectivity while enabling evasion, as evidenced in multiple Omicron sublineages (82, 83). Antibody cocktails targeting multiple spike epitopes have also exhibited diminished efficacy due to concurrent substitutions at those sites. For example, the REGEN-COV cocktail (casirivimab/imdevimab) recognizes two distinct RBD epitopes, but Omicron lineages (e.g., BA.1) accumulated mutations such as K417N and E484A that substantially reduced binding to both components (35, 84). Similarly, suptavumab, an mAb targeting antigenic site V on the RSV F protein, failed in a phase III trial due to prevalent RSV−B strains bearing two amino acid substitutions (L172Q, S173L) in its epitope (85). In summary, the long−term durability of single−epitope mAbs depends on the mutational constraints of the targeted site; such mAbs may retain efficacy against structurally rigid, conserved epitopes (e.g., RSV prefusion F site Ø or the influenza HA stem), whereas they are more vulnerable to evasion by viruses exhibiting high epitope plasticity, such as SARS−CoV−2 (86, 87).

### 2.4 Fc effector functions augment but cannot substitute for neutralization

**Figure 2:**
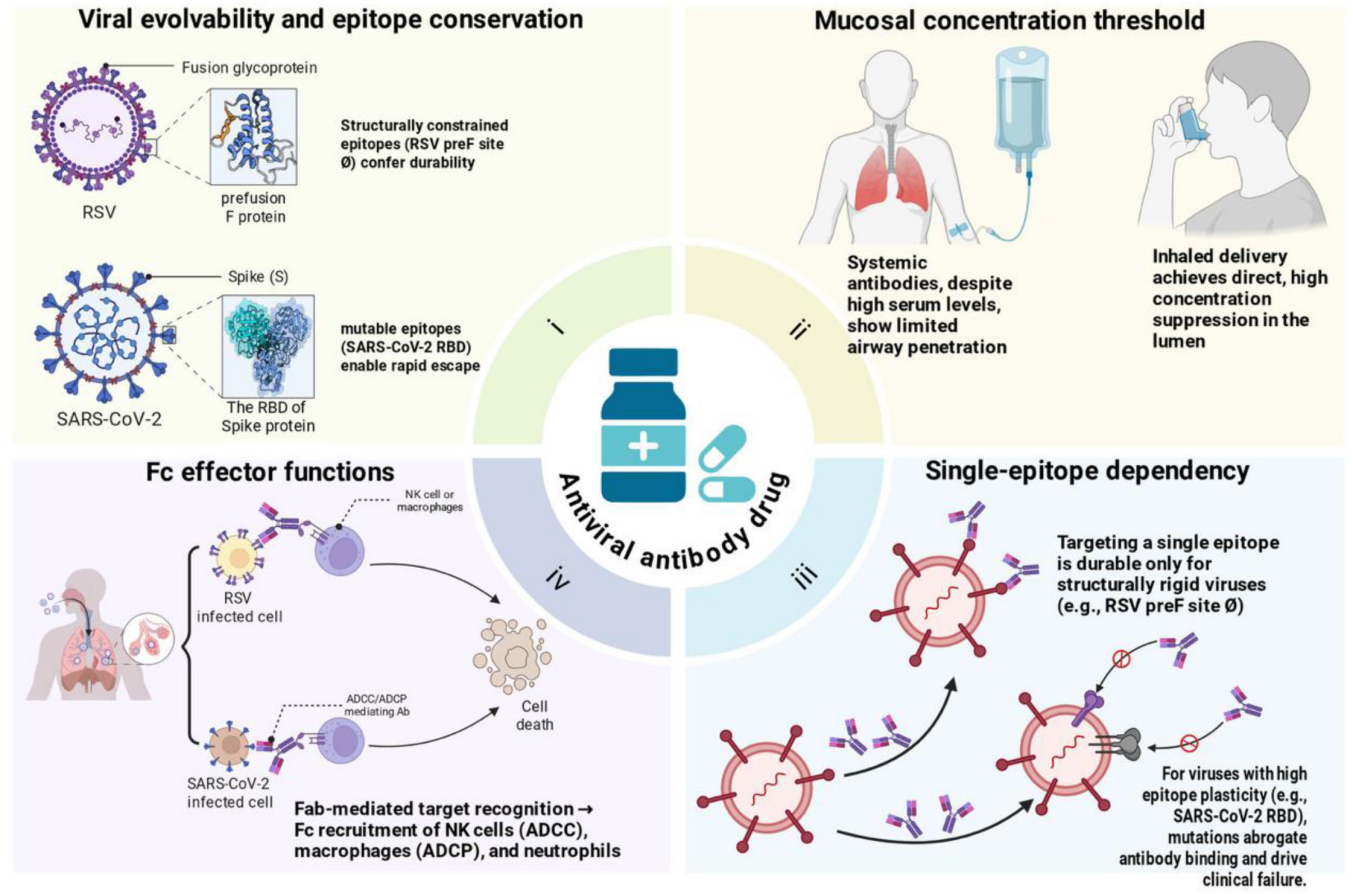
Key determinants of antibody protection against respiratory viruses. The durability of protection is governed by four interrelated factors. **(i)** Viral evolvability and epitope conservation: structurally constrained epitopes, such as RSV preF site Ø, confer long-term susceptibility to neutralizing antibodies due to their marked sequence stability. In contrast, the SARS-CoV-2 spike protein continually remodels the chemical architecture of its antibody epitopes through mutation—thereby undermining the durability of monoclonal antibody therapies—whereas the prefusion-specific site Ø of RSV remains highly conserved, preserving viral vulnerability to neutralization. **(ii)** Mucosal concentration threshold: Systemic administration achieves high serum antibody concentrations but results in limited antibody penetration across the epithelial barrier into the airway lumen. In this setting, rapidly replicating viruses can still initiate breakthrough replication despite circulating antibodies. In contrast, inhaled delivery enables direct antibody deposition within the airway lumen, achieving locally high concentrations that effectively suppress viral replication—even at reduced doses. **(iii)** Single-epitope dependency: Monoclonal antibodies targeting a single epitope are vulnerable to viral escape: mutations at the targeted site can abrogate binding and confer resistance. This vulnerability is context-dependent. Structurally rigid viruses like RSV, which rely on conserved fusion machinery (e.g., prefusion F site Ø), tolerate few escape mutations without compromising fitness, enabling sustained antibody efficacy. In contrast, viruses with high epitope plasticity, such as SARS-CoV-2, permit independent mutations across multiple sites. When antibody binding is disrupted, escape variants rapidly outcompete susceptible strains, rendering single-epitope antibodies clinically ineffective. **(iv)** Fc effector functions: Antibody Fab fragments bind virus or infected cells, while the Fc domain recruits NK cells (ADCC via granzyme/perforin), macrophages (ADCP), and neutrophils to mediate viral clearance. Image created with Biorender.com, with permission.

Beyond neutralization, IgG antibodies can engage Fcγ receptors on immune cells to mediate effector functions, including antibody-dependent cellular cytotoxicity (ADCC), antibody-dependent cellular phagocytosis (ADCP), and complement activation (88–90). These Fc-mediated functions can enhance viral clearance and contribute to protection *in vivo*, particularly once infection is established (91, 92). For example, Fc engineering of anti-SARS-CoV-2 antibodies to selectively engage activating Fcγ receptors has been shown to significantly improve protective efficacy in animal models, such as hamsters and mice, by reducing viral burden and preventing disease progression (93). Moreover, studies in mice have demonstrated that vaccine-elicited serum, including that induced by mRNA-1273, loses substantial protective activity against Omicron variants (e.g., BA.5) when FcγR interactions are disrupted through genetic knockout, highlighting the synergistic role of neutralizing antibodies with immune effector mechanisms (94, 95) (**Figure 2**).

However, Fc functions are insufficient to rescue antibodies whose neutralizing activity has been compromised by viral escape mutations (50, 96). In the Omicron era, next-generation COVID-19 monoclonal antibodies engineered for enhanced Fc potency exhibited no therapeutic benefit once RBD mutations abolished Fab-mediated binding, as observed in pseudovirus neutralization assays and animal challenge models (82, 97). In other words, Fc effector pathways require initial antigen recognition by the antibody variable region; once neutralization is abrogated (as with Omicron escape mutants), Fc engagement is rendered ineffective (98, 99). Estimates from murine models suggest that Fc−mediated contributions may account for less than 20% of overall prophylactic efficacy against SARS−CoV−2, though this fraction can vary with viral dose, route, and host genetics (91, 94).Thus, while Fc optimization can augment antibody effectiveness and potentially reduce the required dose for protection, it does not provide a reliable safeguard against antigenic escape (100). Neutralization remains the essential prerequisite for antiviral antibody efficacy.

## 3 RSV: the first respiratory virus controlled at population scale

### 3.1 Why RSV is biologically suited for antibody prophylaxis

**Figure 3:**
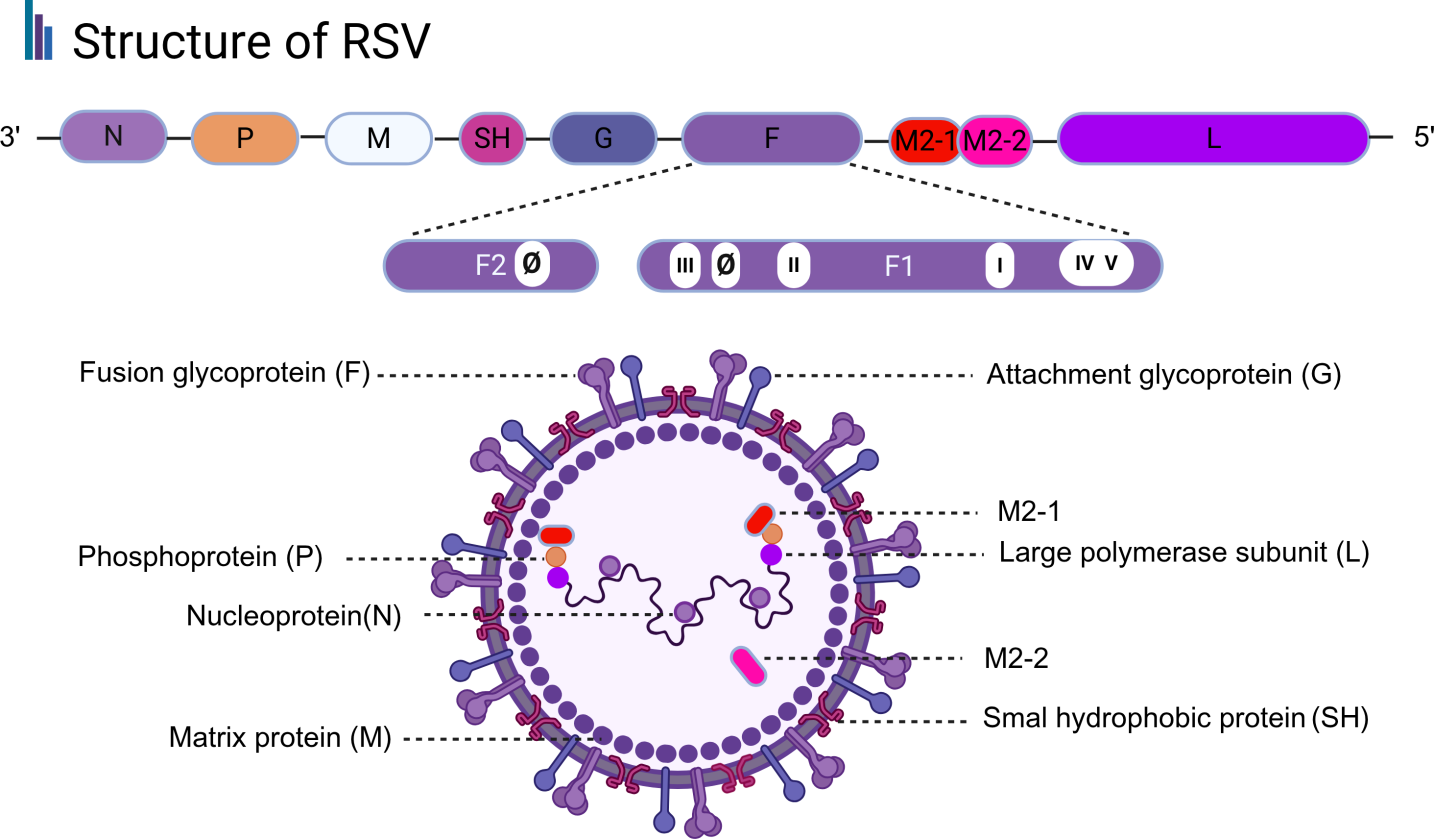
Structure and genome of RSV virus. Schematic of RSV particle showing envelope glycoproteins F, G, and SH; prefusion F trimer with six major antigenic sites highlighted; site Ø (target of nirsevimab) is shown. Image created with Biorender.com, with permission.

RSV exhibits structural and evolutionary features that render it particularly amenable to mAb−based prophylaxis (60, 101). RSV is an enveloped, negative−sense single−stranded RNA virus classified within the Pneumoviridae family. Its genome encodes 11 proteins, including the surface glycoproteins F and G, which are indispensable for host cell invasion: G facilitates viral docking to the respiratory epithelium, while F governs membrane fusion (102, 103). The F protein exists in a metastable prefusion conformation that undergoes dramatic rearrangement to drive fusion (57). Key neutralization epitopes, including site Ø, are exposed only on the prefusion state and are highly conserved across RSV−A and RSV−B strains over decades (58, 81).Mutations in these regions often impair viral fitness, limiting antigenic drift at functionally essential sites (80) (**Figure 3**).

**Figure 4:**
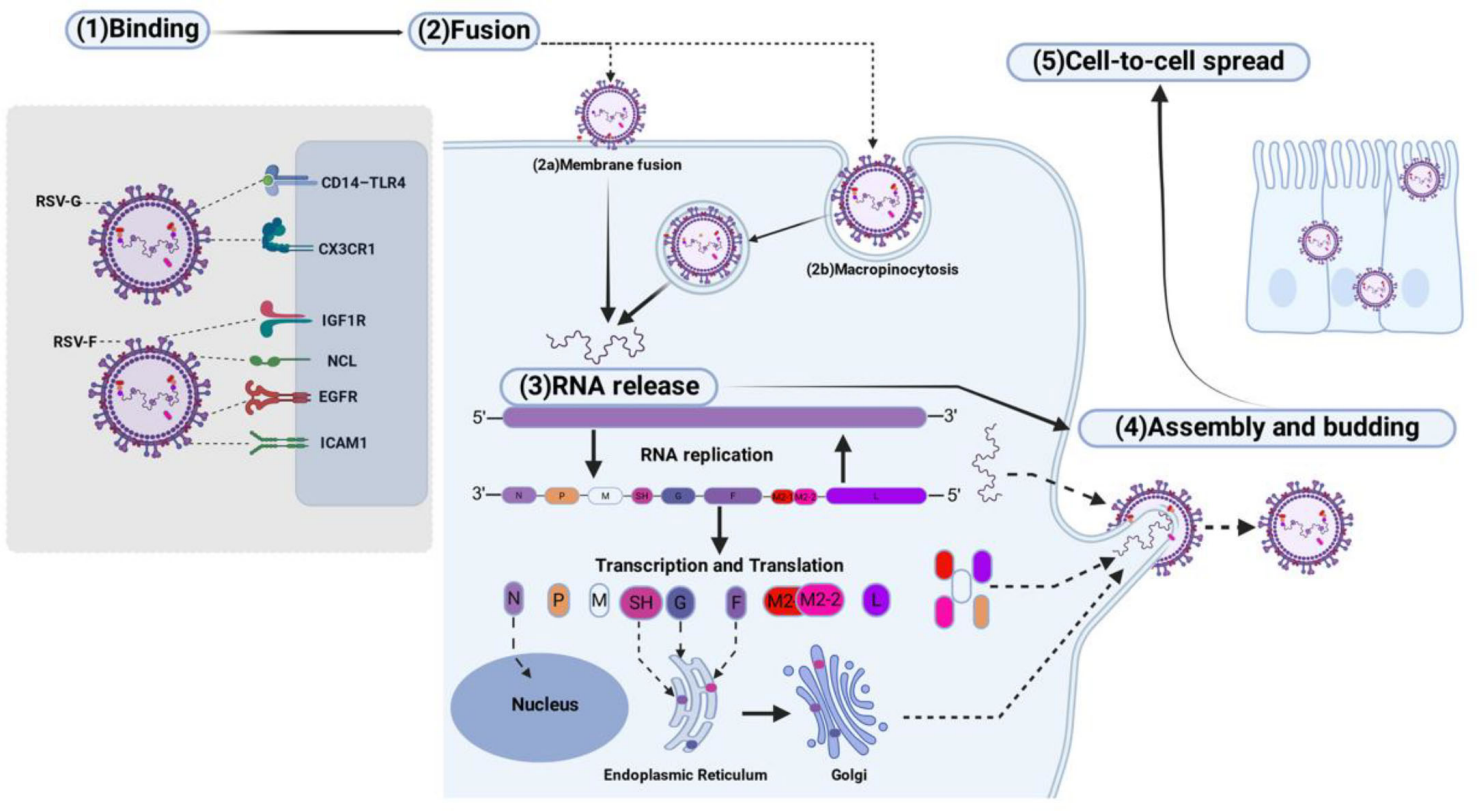
Schematic of RSV infection in host epithelial cells. Viral particles enter via direct fusion or macropinocytosis following engagement of G protein with receptors (CX3CR1, CD14–TLR4) and F protein with (IGF1R, NCL, EGFR, ICAM1). After uncoating, RNA undergoes replication, transcription, and translation. N protein traffic through the nucleus, whereas structural proteins assemble at the plasma membrane; other viral components reach the surface via free ribosomes. Assembly into filaments is followed by membrane fission for particle release.

RSV entry into host epithelial cells occurs either directly at the plasma membrane or via macropinocytosis, initiated by engagement with distinct cellular receptors. The G protein binds to molecules such as CX3CR1, heat shock proteins (HSPs), and the CD14-TLR4 complex, whereas the F protein interacts with IGF1R, nucleolin (NCL), EGFR, and ICAM1. Following internalization, the viral genome is released and uncoated, enabling subsequent replication, transcription, and translation. The newly synthesized proteins are differentially trafficked: the non-structural proteins NS1 and NS2 transit through the nucleus, while structural proteins are processed via distinct pathways. The envelope glycoproteins F, G, and SH are directed to and incorporated into the plasma membrane, whereas other viral components reach the cell surface via free ribosomes. At the membrane, RSV assembles into filamentous structures that are ultimately released through a membrane fission event (24, 102, 104) (**Figure 4**).

### 3.2 Nirsevimab: transformative protection

Nirsevimab, a long−acting monoclonal antibody targeting the prefusion F protein of respiratory syncytial virus (RSV), has demonstrated robust efficacy in preventing severe RSV disease in infants (105). In pivotal randomized controlled trials, a single dose administered at the beginning of the RSV season provided sustained protection for up to 150 days, reducing medically attended RSV−associated lower respiratory tract infections (LRTI) by 70.1% (2.6% vs. 9.5%) and hospitalization risk by 78.4% (0.8% vs. 4.1%) (106). Another trial involving 1490 infants reported a 74.5% reduction in medically attended RSV−LRTI (1.2% vs. 5.0%) and a 62.1% reduction in hospitalization risk (0.6% vs. 1.6%) (24). A large−scale randomized trial (N = 8,058) further confirmed an 83.2% relative risk reduction in RSV−LRTI hospitalizations (0.3% vs. 1.5%) and a 75.7% reduction in very severe RSV infections (107). Pooled analysis from phase 2b and MELODY trials (N = 2,350) showed a 79.5% reduction in medically attended RSV−LRTI and 77.3% reduction in hospitalizations (108). In a trial with 8,057 infants, nirsevimab demonstrated 82.7% efficacy (95% CI 67.8–91.5) in preventing RSV−LRTI hospitalization during the 180−day follow−up (109).

Real−world evidence consistently supports these findings. In a study of 284 eligible children, nirsevimab showed 88.2% effectiveness against RSV−associated acute respiratory infection hospitalization (110). A population−based cohort study in Catalonia (N = 26,525) reported 87.6% protection against RSV bronchiolitis hospitalization and 90.1% against ICU admission (111). US surveillance data indicated 90% protection against RSV−associated hospitalizations (112). Another real−world analysis (N = 10,259) documented 82.0% effectiveness against RSV−LRTI hospitalization and 86.9% protection against severe RSV requiring oxygen (21).These data collectively establish nirsevimab as a transformative, single−dose intervention that substantially reduces the burden of severe RSV disease in infants, with protection levels consistently exceeding 75% against hospitalization and critical outcomes (113).

### 3.3 Why nirsevimab succeeded when others failed

The success of nirsevimab can be attributed to a convergence of strategic design choices that align with the four determinants outlined above. First, it targets the structurally conserved prefusion F site Ø, an epitope critical for viral fusion and intolerant to mutation—unlike the more variable region targeted by the previously unsuccessful suptavumab (114–116). Second, engineered YTE (“LS”) mutations confer an extended half−life (~60–70 days in infants), enabling sustained protection over a typical RSV season from a single dose (108, 109, 117, 118). Third, nirsevimab exhibits exceptionally high neutralization potency, with an IC_50_ approximately 50-100-fold lower than that of palivizumab (108, 114, 118). This allows effective viral neutralization even at the low concentrations achieved in lung ELF, a pharmacokinetic bottleneck that has limited many systemically administered antibodies. Together, these attributes—target conservation, prolonged durability, and high intrinsic potency—constitute a synergistic foundation for population−level effectiveness (24, 107, 114, 118).

### 3.4 Next-generation RSV antibodies

Building on this success, the RSV prophylaxis pipeline continues to evolve. Clesrovimab, approved by the FDA in 2025 as a second long−acting mAb for RSV prophylaxis in infants, targets the highly conserved antigenic site IV of the RSV F protein (99.8% conserved across >11,000 RSV A/B sequences) (71, 119). Engineered with Fc−YTE mutations, it exhibits an extended half−life of approximately 45 days in infants and 73–91 days in adults (120). In the Phase 3 CLEVER trial, a single 105 mg intramuscular dose reduced medically attended RSV−associated lower respiratory tract infections from 6.5% to 2.6% (relative efficacy 60.4%) and RSV−related hospitalizations by 84.2% over 150 days, with a safety profile comparable to placebo (121). Pharmacokinetic studies in adults showed a nasal epithelial lining fluid−to−serum concentration ratio of 1:69–1:30 (1.4-3.3%), supporting its favorable mucosal distribution (71). Recommended by the Advisory Committee on Immunization Practices as an alternative to nirsevimab, clesrovimab provides passive immunization for infants under 8 months of age not protected by maternal vaccination (122). Meanwhile, next-generation platforms such as mRNA-encoded antibodies, nanobodies, and inhalable formulations are under active investigation (123–125). These approaches aim to further enhance mucosal delivery, enable rapid sequence updates in response to viral evolution, and improve accessibility and ease of administration.

In summary, nirsevimab exemplifies how rational antibody design—centered on epitope conservation, pharmacokinetic optimization, and high neutralization potency—can overcome the biological and pharmacological barriers that have historically limited the effectiveness of passive immunization against respiratory viruses. Its success provides a validated template for the development of future antibody−based interventions aimed at achieving durable, population−wide protection against mucosal pathogens.

## 4 SARS-CoV-2: collapse of neutralizing antibodies

Coronaviruses—and SARS−CoV−2 in particular—are RNA viruses with high mutation rates and remarkable structural plasticity, enabling continuous antigenic evolution that rapidly erodes the efficacy of antibody therapeutics targeting immunodominant epitopes (60, 126). The rise and fall of COVID−19 mAbs from 2020 to 2025 is unprecedented in speed and scale (127, 128). In just a few years, an entire class of lifesaving drugs went from highly effective to obsolete, as SARS−CoV−2’s evolution outran our best interventions (129).

### 4.1 Evolution of the receptor-binding domain and antibody resistance

The RBD of the SARS−CoV−2 spike protein has exhibited substantial mutational changes, contributing to reduced efficacy of neutralizing mAbs over time (130, 131). During 2020 and early 2021, mAbs targeting the spike protein, such as casirivimab/imdevimab (REGEN−COV) and sotrovimab, maintained activity against variants like Alpha and Delta, which typically featured limited RBD mutations (132–134). The emergence of Omicron in late 2021, characterized by more than 15 RBD mutations and over 30 spike mutations overall, was associated with marked reductions in neutralization potency for many mAbs (135, 136). Mutations such as K417N, E484A, Q493R, and N501Y were linked to diminished binding for antibody cocktails and single agents, leading to partial or complete resistance in variants like BA.1. Subsequent Omicron sublineages, including XBB.1.5, BQ.1.1, BA.2.86, and JN.1, accumulated additional convergent mutations (e.g., F456L, A475P, Q493E), further eroding antibody activity, with laboratory studies reporting over 100-fold reductions in neutralization against these subvariants compared to ancestral strains. Epistatic interactions among mutations, such as those in XBB (R346T, K444T, L452R, F486S), have been implicated in enhanced immune evasion, rendering many single antibodies ineffective (137, 138). By late 2024, authorized mAbs, including bamlanivimab, REGEN−COV, bebtelovimab, and Evusheld, showed limited or no activity against predominant variants, highlighting the role of antigenic drift in diminishing the utility of these therapies (139, 140).

### 4.2 Timeline of reduced efficacy (2020–2025)

**Figure 5:**
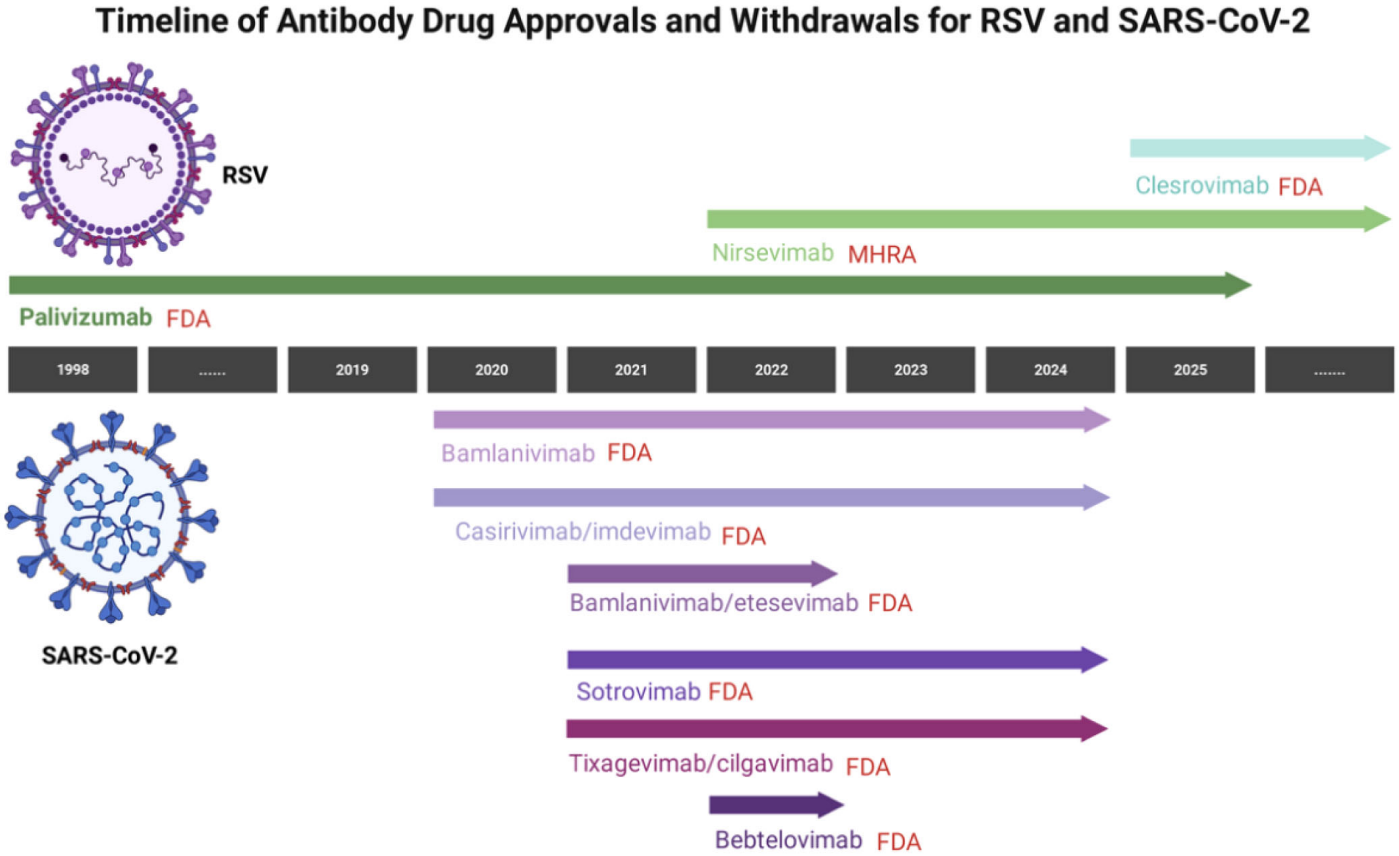
Timeline of neutralizing antibody efficacy against SARS−CoV−2 variants (2020–2025). Shaded bars indicate periods of authorization; crosses mark loss of activity due to variant emergence. Image created with Biorender.com, with permission.

The development and subsequent limitations of neutralizing mAbs for COVID−19 can be delineated across distinct phases. From 2020 to 2021, these agents showed clinical benefits, with trials indicating reductions in hospitalization and severe disease progression of approximately 70% or more when administered early to high−risk patients (128, 129). Multiple products received emergency use authorizations worldwide by late 2021 (139, 140). The Omicron surge in late 2021–2022 led to loss of activity for initial agents; for instance, bamlanivimab/etesevimab and REGEN−COV were revoked due to inefficacy against BA.1. Sotrovimab served as a temporary alternative but was deauthorized following reduced neutralization against BA.2 in spring 2022 (137, 141). In 2023, further Omicron diversification affected remaining options: Evusheld’s authorization was revoked in early 2023 owing to variants like BQ and XBB with mutations such as V445P and R346T (142). Bebtelovimab, active against BA.2/BA.5, lost utility against BF.7 and XBB by mid-2023. Efforts to develop antibodies targeting more conserved sites were pursued, but rapid viral changes limited their advancement (141, 142). By late 2024, regulators, including the FDA, revoked authorizations for remaining products like REGEN-COV, sotrovimab, Evusheld, and bebtelovimab, citing high prevalence of non-susceptible variants (141, 143). As of 2025, no monoclonal antibodies hold authorization for COVID-19 treatment or prevention globally, reflecting challenges in adapting to evolving variants despite initial rapid development successes (144) (**Figure 5**).

### 4.3 Why antibodies failed: integration of the four determinants

The failure of SARS−CoV−2 mAbs can be directly mapped onto the four determinants: (i) Epitope plasticity: The RBD readily accumulated mutations that abolished binding of all therapeutic mAbs, while maintaining or increasing ACE2 affinity (47, 59–63). (ii) Insufficient mucosal concentrations: Systemic IgG reached ELF levels too low and too late to intercept rapid viral replication (peak viral loads at day 2–4) (143, 145–147). (iii) Single−epitope vulnerability: Even cocktails were overcome by concurrent mutations (e.g., K417N+E484A in BA.1) (35, 84). (iv) Fc functions insufficient: Enhanced Fc effector activity could not compensate for loss of Fab binding (96, 148). These elements collectively illustrate that conventional systemic mAbs are ill−suited for viruses with high antigenic drift and acute mucosal replication.

## 5 Challenges and potential pathways forward

The contrasting outcomes of antibody strategies for RSV and SARS−CoV−2 highlight limitations in current systemic approaches for respiratory virus prophylaxis. Refinements such as extended half−life or Fc modifications provide marginal improvements but do not address core barriers (71, 149, 150). Emerging strategies focus on two complementary avenues.

### 5.1 Persistent challenges in systemic IgG administration

Conventional mAbs face constraints that may limit efficacy against many respiratory viruses. These include: (a) Limited penetration into epithelial lining fluid (ELF), where intravenous or intramuscular IgG attains concentrations often below 1% of serum levels due to diffusion barriers (71, 151, 152). (b) Susceptibility to viral evolution: Single or fixed−cocktail mAbs have narrow activity spectra against antigenically drifting RNA viruses (153–155). (c) Modest Fc contribution in prophylaxis: Neutralization is paramount; Fc functions cannot rescue lost binding (156). (d) Narrow therapeutic window: Early administration is critical but logistically challenging in acute infections (152, 157). These challenges necessitate alternative paradigms for viruses like influenza or emerging coronaviruses.

### 5.2 Emerging strategies: mucosal delivery and mRNA−encoded antibodies

Two promising pathways are being actively explored to overcome the limitations of systemic mAbs.

#### 5.2.1 Inhaled or intranasal antibody delivery

**Figure 6:**
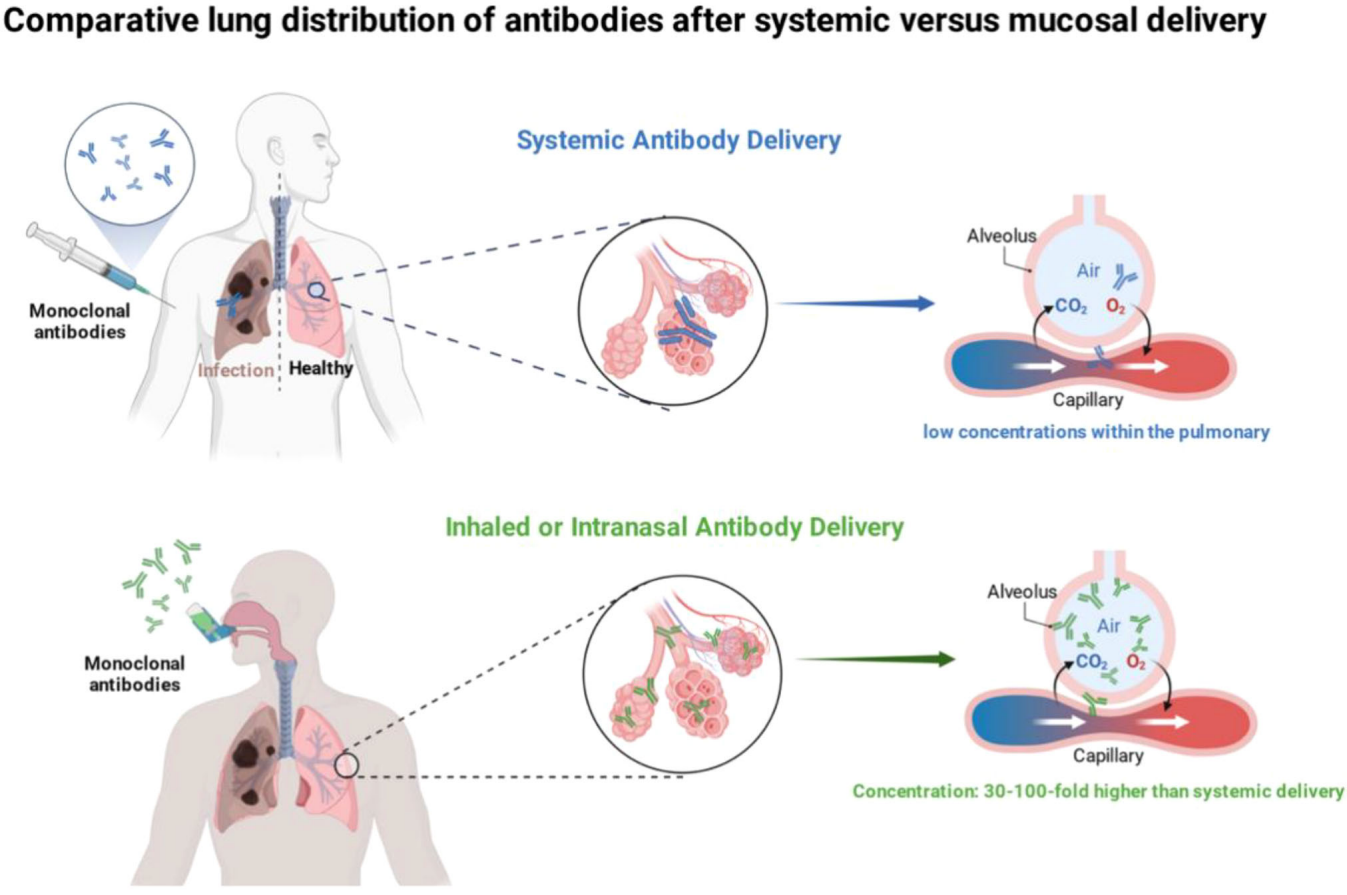
Pharmacokinetic advantage of inhaled vs. systemic antibody delivery. Simulated ELF concentrations after intravenous (blue) and inhaled (green) administration, inhaled delivery achieves rapid, high local concentrations with lower systemic exposure. Image created with Biorender.com, with permission.

Mucosal administration via inhalation or nasal spray can achieve ELF concentrations 30− to 100−fold higher than systemic routes with lower doses (76, 151). Phase 1 trials of inhaled anti−SARS−CoV−2 antibodies reported ELF levels ~40 times serum values; inhaled doses at ~10% of intravenous yielded up to 100−fold higher lung exposure (77). This facilitates localized neutralization with minimal systemic effects. Formats like dimeric IgA enhance mucosal efficacy through antigen cross−linking and protease resistance (158). RSV applications involve inhaled cocktails, with phase 1 data confirming activity retention and distribution (148). For COVID-19, inhaled mAbs in trials show safety and viral load decreases (77, 159). Challenges include stability, dosing consistency (especially in pediatrics), clearance mechanisms, and regulatory pathways for inhaled biologics (160, 161). Successful resolution could enable home−based use, improving accessibility during outbreaks. Inhalable formats may also provide interim protection during emerging pandemics while vaccines are being developed (159) (**Figure 6**).

#### 5.2.2 mRNA−encoded antibodies

Nucleic acid platforms like mRNA facilitate *in vivo* antibody synthesis, offering adaptability, scalability, and targeted delivery (153, 162, 163). Sequences can be updated rapidly to match circulating variants. Lung−targeted lipid nanoparticles in mice produce high ELF antibody titers, protecting against Beta and Omicron (164, 165). This provides passive immunity with sustained production independent of host immunity (166). Programs by Moderna (mRNA−1944 for chikungunya) and BioNTech (BNT−141 for COVID−19) are advancing toward clinical trials (164, 167, 168). Synthetic production may lower costs compared to protein mAbs. Challenges include ensuring correct folding and assembly (e.g., for IgA), mitigating immunogenicity, and achieving organ−specific targeting. Aerosolized lipid nanoparticles are under investigation for respiratory delivery (167–171). Regulatable vectors could control expression duration. Trials are anticipated, potentially for seasonal boosters encoding influenza−neutralizing cocktails in high−risk groups.

### 5.3 Integrated future approaches

Inhaled and mRNA−encoded methods may be combined for enhanced effect. For example, inhaled IgG could provide immediate prophylaxis, while nasal mRNA delivery enables sustained local production of variant−adapted antibodies (172–174). Bispecific antibodies could address virus families via these routes, enabling pan-coronavirus immunity (175–177). Simplified delivery devices (e.g., dry powder inhalers) and tailored regimens for immunocompromised patients could broaden accessibility and adherence. Ultimately, these innovations may blur the lines between drugs, gene therapies, and vaccines, enabling agile responses to viral threats.

## 6 Conclusion

The starkly divergent outcomes of antibody−based interventions against RSV and SARS−CoV−2 establish a fundamental paradigm for the future of antiviral mAb development. Success, exemplified by nirsevimab, is predicated on the precise alignment of three non−negotiable biological and pharmacological pillars: (1) targeting a structurally constrained, evolutionarily conserved viral epitope; (2) achieving and sustaining neutralizing antibody concentrations at the primary site of mucosal infection; and (3) matching the intervention kinetics to the replication dynamics of the pathogen. RSV’s conserved prefusion F protein site Ø, coupled with a long−acting, high−potency mAb design, fulfilled these criteria, enabling durable, population−level protection.

In contrast, the rapid collapse of SARS−CoV−2 neutralizing mAbs resulted from the misalignment of these same principles: a highly plastic RBD under intense immune selection, insufficient mucosal antibody levels from systemic delivery during early, high−titer viral replication, and an evolutionary pace that outstripped therapeutic development. This experience decisively concludes that for respiratory viruses with high antigenic turnover or acute mucosal pathogenesis, conventional intravenous/intramuscular mAbs are an unsustainable solution.

The path forward requires a paradigm shift. Future strategies must transcend these limitations by design. Innovations in mucosal delivery (e.g., inhaled mAbs) and rapid−adaptation platforms (e.g., mRNA−encoded antibodies) represent the most promising avenues to ensure adequate frontline concentration and resilience against viral escape. By rigorously applying the mechanistic lessons distilled from these two landmark cases—prioritizing epitope conservation, guaranteeing mucosal bioavailability, and respecting viral evolutionary kinetics—the next generation of antibody therapeutics can be rationally engineered to deliver durable protection against a broader spectrum of respiratory threats.
